# Poisoning with Sulphuric Acid—Death of Mr. Ledyard

**Published:** 1828-05

**Authors:** 


### II. ANALYTICAL.

3. *Poisoning with Sulphuric Acid.* In the eloquent life of Mr. Ledyard, the American traveller, just published from the pen of Mr. Sparks, it is stated that the Traveller, when preparing to depart from Cairo for the interior of Africa, "was seized with a bilious complaint, which he thought to remove by the common remedy, *Vitriolic Acid.* Whether this was administered by himself, or by some other person, is not related; but the quantity taken was so great, as to produce violent and burning pains that threatened to be fatal, unless immediate relief could be procured. This was attempted by a powerful dose of *tartar emetic.* But all was in vain. The best medical skill in Cairo was called to his aid without effect." That he should have died under such a method of treatment, is not surprising. It is not our purpose, however, to rail against our Egyptian brethren, but to connect with the name and fate of our distinguished countryman, a catalogue of the proper antidotes for the poison which occasioned his death; that we may contribute our mite towards their general dissemination.

When the liquor called Sulphuric Acid, Vitriolic Acid and Oil of Vitriol, or the medicine formed from it and denominated Elixir Vitriol, is taken to excess, tartar emetic should not be administered, for it would be decomposed; and, if it were not, the apprehended mischief would be perpetrated before it could operate. An alkaline agent is that on which we should rely; and when one is not at hand, another should be taken. Calcined Magnesia, according to Orfila, is the best, and may be taken by the table spoonful. The lump or carbonated Magnesia will answer very well. The supercarbonate and the carbonate of Soda; the supercarbonate of Potash, or sal æratus, the carbonate of potash or salt of Tartar, and a ley made of common wood ashes, are equally good. In the absence of all these, powdered Chalk, Lime-water, or Quick lime, if within reach, may be administered. Finally, should none of these be attainable, at the moment, the contents of the hartshorne or the smelling bottle (carbonate of Ammonia) may be emptied into the patient's stomach. If the acid were taken in a concentrated state, it would be useful to drink water freely; but, that its union with the poison, would develope a degree of heat, that might more than counterbalance the good effects of the dilution. Meanwhile, if vomiting can be excited by irritating the throat, it should be done; or the stomach may, perhaps, be emptied by strong pressure with the fists applied over that organ, the patient being placed with his face downwards, and his head and shoulders considerably lower than the trunk of his body; or the stomach pump may be used.

The same antidotes will be proper when the Nitric, Muriatic, or any other strong acid, has been taken.

The experienced physician will, we trust, excuse this enumeration, seeing that it may, possibly, be useful to the younger members of the profession; and finding its way to the people at large, may enable them, occasionally, to save an unfortunate person, who might perish before medical advice could be obtained.

